# Call a spade a spade: taxonomy and distribution of *Pelobates*, with description of a new Balkan endemic

**DOI:** 10.3897/zookeys.859.33634

**Published:** 2019-07-02

**Authors:** Christophe Dufresnes, Ilias Strachinis, Elias Tzoras, Spartak N. Litvinchuk, Mathieu Denoël

**Affiliations:** 1 Laboratory for Conservation Biology, University of Lausanne, 1015 Lausanne, Switzerland; 2 Hintermann & Weber SA, Avenue des Alpes 25, 1820 Montreux, Switzerland; 3 Department of Animal and Plant Sciences, University of Sheffield, Alfred Denny Building, Western Bank, S10 2TN Sheffield, United Kingdom; 4 School of Biology, Aristotle University of Thessaloniki 54124 Thessaloniki, Greece; 5 26442 Patra, Achaia, Greece; 6 Institute of Cytology, Russian Academy of Sciences, Tikhoretsky pr. 4, St. 194064 Petersburg, Russia; 7 Department of Zoology and Physiology, Dagestan State University, Gadzhiyev str. 43-a, 336700 Makhachkala, Dagestan, Russia; 8 Laboratory of Fish and Amphibian Ethology, Behavioural Biology Group, Freshwater and OCeanic science Unit of reSearch (FOCUS), University of Liège, Liège, Belgium

**Keywords:** Amphibian, Palearctic, *
Pelobates balcanicus
*, *
Pelobates balcanicus chloeae
*, *
Pelobates vespertinus
*, Pelobatidae, phylogenomics, phylogeography, spadefoot toad

## Abstract

The genomic era contributes to update the taxonomy of many debated terrestrial vertebrates. In an accompanying work, we provided a comprehensive molecular assessment of spadefoot toads (*Pelobates*) using genomic data. Our results call for taxonomic updates in this group. First, nuclear phylogenomics confirmed the species-level divergence between the Iberian *P.cultripes* and its Moroccan relative *P.varaldii*. Second, we inferred that *P.fuscus* and *P.vespertinus*, considered subspecies until recently, feature partial reproductive isolation and thus deserve a specific level. Third, we evidenced cryptic speciation and diversification among deeply diverged lineages collectively known as *Pelobatessyriacus*. Populations from the Near East correspond to the Eastern spadefoot toad *P.syriacus* sensu stricto, which is represented by two subspecies, one in the Levant (*P.s.syriacus*) and the other in the rest of the range (*P.s.boettgeri*). Populations from southeastern Europe correspond to the Balkan spadefoot toad, *P.balcanicus*. Based on genetic evidence, this species is also polytypic: the nominal *P.b.balcanicus* inhabits the Balkan Peninsula; a new subspecies *P.b.chloeae***ssp. nov.** appears endemic to the Peloponnese. In this paper, we provide an updated overview of the taxonomy and distribution of all extant *Pelobates* taxa and describe *P.b.chloeae***ssp. nov.**

## Introduction

The revolution initiated by high-throughput sequencing techniques has reached the field of phylogeography (Coates et al. 2018), where it lifts the veil on cryptic species and solves long-term taxonomic issues (e.g. Rodriguez et al. 2017; Psonis et al. 2018; Dufresnes et al. 2018, 2019a). We conducted such study in spadefoot toads from the monotypic family Pelobatidae Bonaparte, 1850 (genus *Pelobates* Wagler, 1830) endemic to the Western Palearctic (Dufresnes et al. 2019b). These grassland species typically inhabit soft (e.g. sandy) soils with freshwater ponds for breeding and have a semi-fossorial lifestyle, thanks to well-known adaptations such as metatarsal spades (to dig themselves in) and a strongly ossified skull (to dig themselves out) (Székely et al. 2017; Dufresnes 2019). They are threatened in many parts of their fragmented ranges due to land-use changes, wetland destruction, pollution, species introduction, and ongoing changes in climate, which already led to population extinctions and contractions of geographic ranges (Nyström et al. 2002, 2007; Džukić et al. 2005; Eggert et al. 2006). Mediterranean regions, where most of the diversity is located (Litvinchuk et al. 2013; Dufresnes et al. 2019b), could be particularly threatened (Iosif et al. 2014).

Until recently, *Pelobates* included four recognized extant species. First, the sister taxa *P.cultripes* (Cuvier, 1829) and *P.varaldii* Pasteur & Bons, 1959 are found north and south of the Strait of Gibraltar, respectively (Busack et al. 1985). Second, the western and eastern sister taxa *P.fuscus* (Laurenti, 1768) and *P.vespertinus* (Pallas, 1771) were long considered subspecies (e.g. Crottini et al. 2007), but their narrow transition is rather consistent with a species level (Litvinchuk et al. 2013). Third, Mediterranean populations from the Near East and the Balkans are commonly referred to as *P.syriacus* Boettger, 1889 and split as two subspecies: *P.syriacussyriacus* in Asia Minor and *P.syriacusbalcanicus* Karaman, 1928 in the Balkans, based on morphological (Uğurtas et al. 2002) and scattered phylogenetic data (Veith et al. 2006; Litvinchuk et al. 2013; Ehl et al. 2019).

Our accompanying paper (Dufresnes et al. 2019b) revisits the evolution of this group, with several taxonomic implications. First, phylogenomics confirmed the old split between *P.cultripes* and *P.varaldii*, previously identified with mtDNA (Garcia-Paris et al. 2003; Veith et al. 2006; Crottini et al. 2007) and allozyme markers (Busack et al. 1985; Litvinchuk et al. 2013). Second, hybrid zone analyses support the conclusions of Litvinchuk et al. (2013) that *P.fuscus* and *P.vespertinus* deserve a specific status. Third, *P.syriacus* represents two cryptic species respectively distributed in the Near East and the Balkans, then corresponding to *P.syriacus* and *P.balcanicus*. Fourth, these species feature deep intraspecific divergence, worthy of subspecific status. This is the case between Levantine and Anatolian/Caucasian populations in *P.syriacus*, and between the northern Balkans and Peloponnese in *P.balcanicus*.

**Figure 1.**
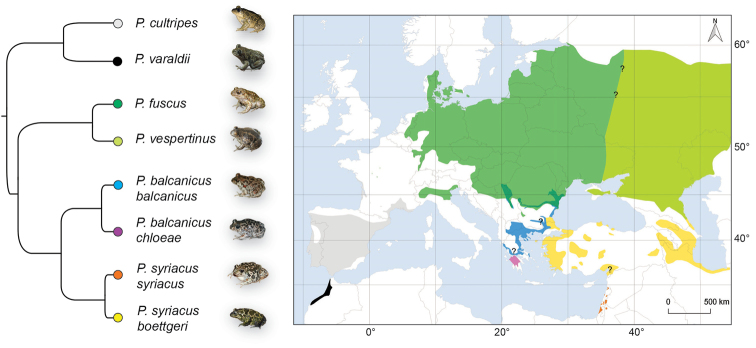
Phylogeny and distribution of *Pelobates* taxa. The tree is adapted from the phylogenomic analysis of Dufresnes et al. (2019b), and the map was built from known records updated with genetic data (see accounts). Note that the distribution of *P.vespertinus* extends further east to Kazakhstan and Siberia. Photo credits: CD (*P.cultripes*, *P.b.chloeae*), SNL (*P.s.boettgeri*), IS (*P.b.balcanicus*), A Sanchez Vialas (*P.varaldii*), A Nöllert (*P.fuscus*), N Suriadna (*P.vespertinus*).

In this paper, we integrate these recent findings into an updated overview of the *Pelobates* radiation, including comparative diagnosis, current taxonomy, distribution, and diversity of the resulting eight extant taxa (Fig. 1). Last but not least, we describe the newly discovered clade from Peloponnese as a subspecies of *P.balcanicus*.

## Material and methods

### Nomenclatural search

In order to attribute names to the newly documented *Pelobates* species and subspecies, we examined nomina available in the literature. To this end, we first referred to the Amphibian Species of the World online database (Frost 2019) and subsequently reviewed all the original references available.

### Diagnosis

We reviewed phenotypic (coloration, morphology) and genetic (genome size, karyotype, and sequence divergence) variation of the considered taxa. Coloration is illustrated by high-quality photographs of known geographic origins, taken by us and credited photographers. Besides detailing general characteristics, we compiled a dataset of snout-vent length (SVL) from published studies (Suppl. material 1, Table S1), consisting of average SVL (computed separately for males and females) from 82 populations, totaling 6,004 individuals at least, and representing all taxa expect the narrowly distributed *P.s.syriacus* and *P.b.chloeae* (Suppl. material 1, Table S1). We report the ranges (minimum-maximum) and average values for each sex separately, and illustrate interpopulation variation by boxplots. We statistically tested differences among taxa and sex by a two way analysis of variance (ANOVA) in *R.* We then performed comparisons between species using a Tukey test. Finally, we tested sex-specific differences within taxa for which measures of both sexes were available in at least five populations, by paired *t*-tests.

We briefly described the karyotype of each taxon based on the literature and further report nuclear DNA content as a proxy to genome size, obtained from flow cytometry. In addition, sequence divergence, available from our phylogeographic study (Dufresnes et al. 2019b), are provided between each pair of taxa, based on mitochondrial (*cyt-b* + 16S, 1.2 kb) and nuclear DNA (63.5 kb of RAD tags).

### Distribution

We detailed the distribution of each *Pelobates* taxon, based on available literature, i.e. national and regional atlas, as well as scientific articles informative of distribution. Boundaries between cryptic taxa were inferred from genetic studies, and thus remain unclear for parapatric ranges for which no molecular survey has been conducted. Distribution layers were originally obtained from the IUCN Red List of Threatened Species (https://www.iucnredlist.org/), and meticulously reshaped with the drawing tools of ArcMap 10.3.

### Description of *Pelobatesbalcanicuschloeae* sp. nov.

In order to describe the new *P.balcanicus* subspecies from southern Greece, we conducted a short fieldwork expedition to the only recently confirmed locality of this taxon, Strofylia meadows in Peloponnese (38.1239°N, 21.3858°E) on December 2018. Collection of live animals was authorized by permit ΑΔΑ: ΩΣΜ34653Π8-9ΣΟ issued by the Greek Ministry of Environment, Energy and Climate Change. *Pelobates* usually breed during spring (March–April) in this area but are active all-year round providing proper weather conditions. A total of 18 individuals could be captured in the evening of December 10^th^, under heavy rains. The largest 12 individuals (putatively adults) were measured for 11 standard morphometric variables, i.e. SVL: snout-vent length; HW: head width; HL: head length; ED: eye diameter; EE: inter-eye distance; NN: inter-nostril distance; EN: eye-nostril distance; ML: metatarsal tubercle length; MH: metatarsal tubercle height; HLL: hind leg length; TTL: tibia + tarsus length. HLL and TTL were measured with a ruler (1 mm precision); all other variables were measured with a digital caliper (0.1 mm precision). For the sake of comparison, only one of us (IS) measured all individuals. Note that we did not discriminate the sex of individuals as it was unclear whether all specimens were adults.

Toads were subsequently released at their place of capture, except for two females that were chosen as holotype and paratype, sent to the Natural History Museum of Crete (NHMC). Our choice for a small type series was bounded by the rarity of this taxon, so far confirmed from a single locality, with unknown population trends.

## Results and discussion

We updated the distributions and taxonomy of Eurasian spadefoot toads (genus *Pelobates*). Following recent molecular results (Dufresnes et al. 2019b), a total of eight extant clades are distinguished. Six of them correspond to species level divergences, given their confirmed or putative reproductive isolation, as inferred from hybrid zone analyses, which make ad hoc tests to evaluate where two lineages stand along the speciation continuum (Singhal and Moritz 2013; Dufresnes et al. 2019b). The remaining intraspecific lineages are accordingly treated as subspecies, because they featured extensive admixture and thus seem to lack reproductive barriers.

From our SVL dataset, there was a significant global effect of species (*P* < 0.001) but not of sex (*P* = 0.08), neither of their interaction (*P* = 0.42) (two way ANOVA). The species effect was mainly due to differences between the large *P.cultripes*, *P.syriacus*, and *P.balcanicus* versus the smaller *P.varaldii*, *P.fuscus*, and *P.vespertinus*: all pairwise comparisons between these two groups were significant (*P* < 0.001), but none within groups (*P* > 0.05) (Tukey test). Females were significantly larger than males in *P.cultripes* (*P* = 0.002, *n* = 16 populations with both sexes), *P.fuscus* (*P* < 0.001, *n* = 21), but not in *P.balcanicus* (*P* = 0.58, *n* = 15) (paired *t*-test). Sample size precluded similar analyses in the remaining taxa.

**Figure 2.**
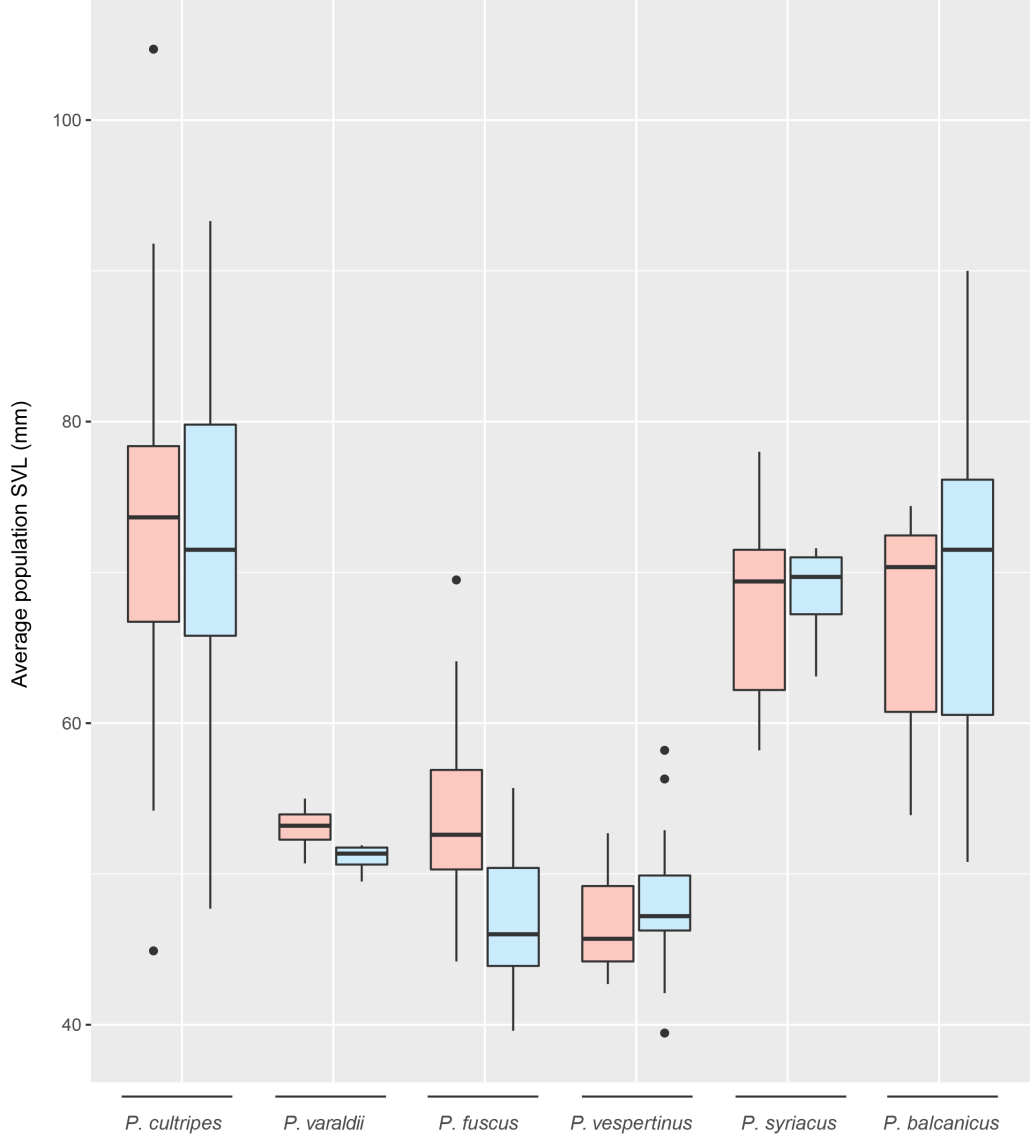
Between-population variation of average size (snout–vent length – SVL) for each *Pelobates* species, measured separately for females (pink) and males (blue). This compiles average size-data from 82 populations, representing at least 6,004 individuals (Suppl. material 1, Table S1). For *P.balcanicus*, it only includes populations from the nominal *P.b.balcanicus*. For *P.syriacus*, it only includes populations from *P.s.boettgeri*.

**Figure 3.**
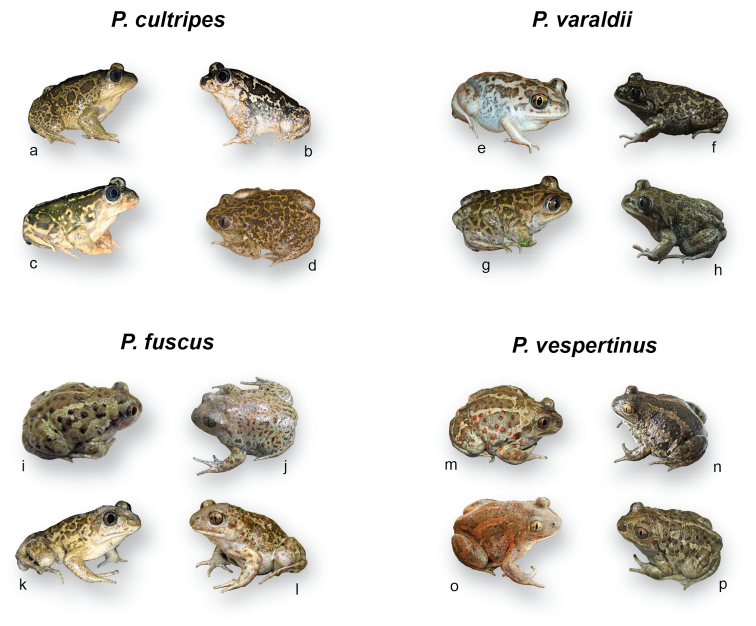
Color variation in *Pelobatescultripes*, *P.varaldii*, *P.fuscus* and *P.vespertinus*. Photo credits and origins as follows **a** CD (Hérault, France) **b, c** CD (Algarve, Portugal) **d** A Sanchez Vialas (Spain) **e** G Martinez (Kenitra, Morocco) **f–h** A Sanchez Vialas (Tanger, Morocco) **i, j** N Suriadna (Ukraine) **k** CD (Wojewodztwo podkarpackie, Poland) **l** A Nöllert (Burgenland, Austria) **m–p** N Suriadna (Ukraine).

**Figure 4.**
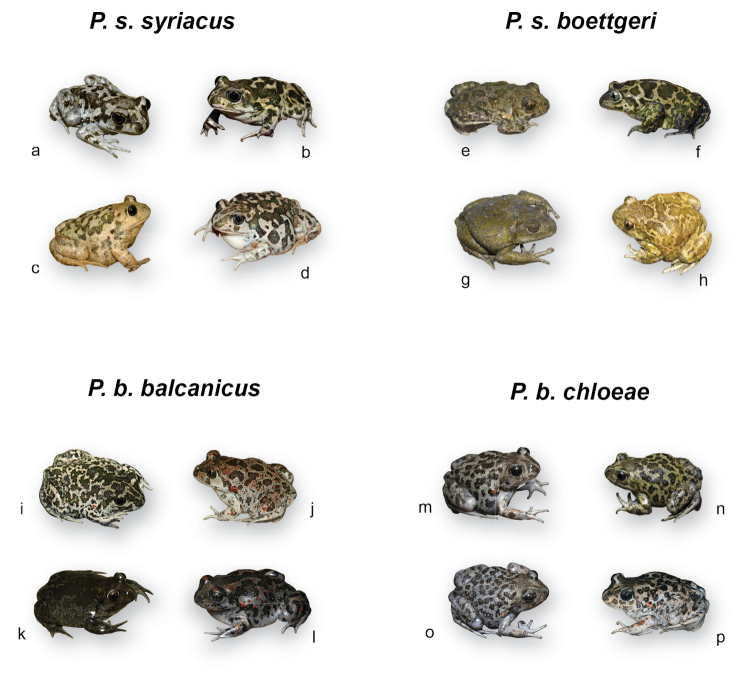
Color variation in *Pelobatessyriacus* and *P.balcanicus*. Photo credits and origins as follows **a, b** G Hamoivitch (Israël) **c** R Winkler (Israël) **d** G Martinez (Israël) **e** IS (Limnos, Greece) **f** SNL (European Turkey) **g** IS (Limnos, Greece) **h** A Nöllert (Dagestan, Russia) **i** MD (Danube Delta, Romania) **j** IS (Thrace, Greece) **k** IS (Macedonia, Greece) **l** IS (Evia, Greece) **m–o** IS (Peloponnese, Greece) **p** CD (Peloponnese, Greece).

The following present accounts for each taxon. We could successfully access the original literature for all but one description, and herein report the primary information as it was published. The only exception is *Pelobatespraefuscus* Khosatzky, 1985, and we rely on Frost (2019) for its information. Phylogeny and distributions of extant *Pelobates* are shown in Figure 1, sizes and color variation are displayed in Figure 2, and Figures 3 and Figures 4, respectively.

### Pelobates cultripes

Taxon classificationAnimaliaAnuraPelobatidae

(Cuvier, 1829)

#### Diagnosis.

The largest *Pelobates* species, *P.cultripes* differs from the other Eurasian spadefoots by metatarsal spades being entirely black and a flat skull. Sizes largely overlap between sexes although males are generally smaller than females (Fig. 2). The background coloration can be yellow, gray, or brown, reticulated by dark patches; it typically lacks orange spots (Fig. 3). Average SVL = 74 mm (range: 32–105 mm) for females (*n* = 16 populations) and 71 mm (34–93 mm) for males (*n* = 17 populations) (Suppl. material 1, Table S1; Fig. 2). The karyotype consists of six large and seven small (i.e. < 6% of total length) pairs of two-armed chromosomes (Morescalchi 1967, 1971; Morescalchi et al. 1977; Schmid et al. 1987; Herrero and Talavera 1988). Large centromeric C-bands appears in pairs 1, 2, 4, 9, and 12; pericentric bands in the short arm of pair 1 and the long arm of pair 8; telomeric bands in the long arms of pairs 1, 2, and 11; the short arm of pair 7 is almost heterochromatic (Herrero and Talavera 1988). Nucleolus organizers (NORs) are in the short arm of pair 7 (Schmid et al. 1987). The nuclear DNA content averages 7.4 pg (Litvinchuk et al. 2013).

#### Taxonomy.

First named ***Ranacultripes* Cuvier, 1829**; holotype: MNHNP 0.4554; type locality: “notre midi”, corresponds to southern France, as noted by Mertens and Müller (1928). Two junior synonyms. ***Ranacalcarata* Michahelles, 1830**; type locality: “prope Malagam” (near Malagam), probably Malaga, Spain; type(s): not mentioned. ***Cultripesprovincialis* Müller, 1832**; type locality: “Provence” (meridional France), France; type(s): not designated, but the author refers to *Ranacultripes* from Paris (MNHN). First mentioned as *Pelobatescultripes* by Tschudi (1838).

#### Distribution.

The species inhabits south-western Europe (0–1770 m elevation a.s.l.) (Sillero et al. 2014; Beja et al. 2009) (Fig. 1). Its main distribution spans across the Iberian Peninsula, where it occurs roughly everywhere in suitable habitats south of the Cantabrian Mountains and Pyrenees (Lizana 1997; Malkmus 2004). It is yet absent from the south-eastern tip of Spain (Lizana 1997). In France, it is present only along the Atlantic coast, from the Landes region to the Loire River, and along the Mediterranean Sea, from the Spanish border to the Var Department, reaching the area of Valence in the Rhone Valley. Some isolates exist also in south-western France (Thirion and Cheylan 2012). IUCN status: Near Threatened (Beja et al. 2009).

#### Diversity.

Combining mtDNA and microsatellite data, Gutiérrez-Rodriguez et al. (2017) identified three closely-related mtDNA haplogroups (see also Crottini et al. 2010) in the southern, western / northwestern, and northeastern parts of the range, which are mirrored by equivalent nuclear clusters that widely admix. Most of the genetic diversity of this species is found in southern ranges, where climate conditions remained stable through the last ice ages (Gutiérrez-Rodriguez et al. 2017).

### Pelobates varaldii

Taxon classificationAnimaliaAnuraPelobatidae

Pasteur & Bons, 1959

#### Diagnosis.

A smaller version of *P.cultripes* (Fig. 2) differing by a few phenotypic features. Unlike *P.cultripes*, the black coloration of the spades is often concentrated on the edges (Pasteur and Bons 1959; Busack et al. 1985). The cranial braincase is high and narrow in *P.varaldii*, while it is low and wide in *P.cultripes* (Pasteur and Bons 1959; Roček 1981). The background coloration can be yellow, gray, and brown, with dark reticulate patches, and the dorsal surface is abundantly covered by orange dots, most pronounced on the eyelids (usually absent in *P.cultripes*; Pasteur and Bons 1959; Beukema et al. 2013; Fig. 3). Males are usually smaller than females (Fig. 2). Average SVL = 53 mm (range: 36–66 mm) for females (*n* = 4 populations) and 51 mm (33–65 mm) for males (*n* = 4 populations) (Suppl. material 1, Table S1; Fig. 2). The karyotype includes six large and seven small pairs of two-armed chromosomes. Large centromeric C-bands appears in the pairs 1, 2, 4, 9, and 12; pericentric bands in the short arms of pair 1 and long arm of pair 8; telomeric bands in the long arms of pairs 1, 2, and 11; the short arm of pair 7 is almost heterochromatic (Herrero and Talavera 1988). The nuclear DNA content averages 7.3 pg (Litvinchuk et al. 2013). As shown in Table 1, *P.varaldii* differs from *P.cultripes* by 6.0% at mtDNA and 0.40% at nuclear DNA (Dufresnes et al. 2019b).

**Table 1.** Pairwise % of genetic differences between *Pelobates* taxa (from the data of Dufresnes et al. 2019b). The estimates below diagonal correspond to mitochondrial DNA (*cyt-b* + 16S, 1.2 kb); the estimates above diagonal correspond to nuclear DNA (63.5 kb of RAD tags).

	*** P. cultripes ***	*** P. varaldii ***	*** P. fuscus ***	*** P. vespertinus ***	*** P. s. syriacus ***	*** P. s. boettgeri ***	*** P. b. balcanicus ***	*** P. b. chloeae ***
*** P. cultripes ***	–	0.40	0.66	0.75	0.72	0.70	0.74	0.73
*** P. varaldii ***	6.0	–	0.83	0.92	0.89	0.88	0.92	0.90
*** P. fuscus ***	10.1	10.0	–	0.13	0.63	0.62	0.65	0.64
*** P. vespertinus ***	9.7	9.6	2.5	–	0.71	0.70	0.74	0.73
*** P. s. syriacus ***	9.1	8.6	9.1	8.9	–	0.01	0.32	0.30
*** P. s. boettgeri ***	9.2	8.9	9.2	9.0	1.7	–	0.31	0.29
*** P. b. balcanicus ***	9.2	8.6	8.5	8.5	7.2	7.0	–	0.02
*** P. b. chloeae ***	9.2	8.2	8.5	8.6	7.7	7.7	2.8	–

#### Taxonomy.

The nomen ***Pelobatesvaraldii* Pasteur & Bons, 1959** is the only one ever proposed for the Moroccan populations of spadefoot toads; holotype: MNHN-RA-1959.1; type locality: Merja Samora, Morocco. The ancient split of *P.varaldii*, dating back to the Messinian Salinity Crisis (5.3 My), supports its specific distinction from *P.cultripes* (Busack et al. 1985; Crottini et al. 2007).

#### Distribution.

It is endemic to north-western Morocco (0–350 m elevation a.s.l.), found along the Atlantic coast, from the south of Tanger to Oualidia, where it is rare (de Pous et al. 2012; Beukema et al. 2013; Frost 2019). IUCN status: Endangered (Salvador et al. 2009).

#### Diversity.

To our knowledge, *P.varaldii* has not been the focus of any phylogeographic or population genetic work.

### Pelobates fuscus

Taxon classificationAnimaliaAnuraPelobatidae

(Laurenti, 1768)

#### Diagnosis.

Small spadefoot characterized by pale grayish metatarsal spades and a domed skull. The webbing of the hindfeet is well developed. Males are smaller than females (Fig. 2). The species can be found in a spectrum of gray, brown, or yellowish colors, but rarely greenish (P. Székely pers. comm.), and features patterns such as stripes or blotches of varying sizes; variable presence of orange dots, from almost absent to very abundant (Fig. 3). In Eastern Europe, it differs from its sister species *P.vespertinus* by most individuals having numerous dark rounded spots on a light dorsum (Suriadna et al. 2016) and lacking a dark stripe between the eyes (Lada et al. 2005). Average SVL = 54 mm (range: 37–78 mm) for females (*n* = 21 populations) and 47 mm (36–65 mm) for males (*n* = 21 populations) (Suppl. material 1, Table S1; Fig. 2). The karyotype consists of seven large and six small pairs of two-armed chromosomes (Mészáros 1973; Schmid et al. 1987; Manilo and Radchenko 2004; Manilo and Manuilova 2013; Suriadna 2014). Centromeric C-bands are obvious in pairs 2, 6, and 7–13 (Schmid et al. 1987). NORs are in the short arm of pair 7 (Schmid 1980, 1982). The nuclear DNA content (calculated from flow cytometry) averages 8.7–9.0 pg (Litvinchuk et al. 2013).

#### Taxonomy.

Originally described as ***Bufofuscus* Laurenti, 1768**; type locality: not specifically designated (“in paludibus, rarissime hospitantur in continenti”, in swamps, rarely on the land); type(s): the specimens depicted by Rösel von Rosenhof (1758: pls XVII, XVIII), expressively cited by Laurenti (1768); although controversial (see Nöllert et al. 2012; Frost 2019), the additional mention of pl. XV (p. 122), a drawing of a dissected *Pelophylax*, could simply be an error. Rösel depicted the amphibians of Germany, and Shaw (1802) accordingly mentioned that Rösel found his specimens in the neighborhood of “Nurenberg” (Nürnberg), Germany, which could then apply as the type locality. Seven junior synonyms. ***Ranaalliacea* Shaw, 1802**; type locality: not specifically designated, but Shaw (1802) refers to Rösel’s toads from Nürnberg, Germany; type(s): the toad illustrated by the author (pl. 41), which may very well corresponds to the amplexed female on the top right of pl. XVII in Rösel von Rosenhof (1758), of identical posture and color patterns. ***Bombinatormarmorata* Sturm, 1828**; type locality: near Penig, Germany; holotype: the frog illustrated by the author. ***Cultripesminor* Müller, 1832**; type locality: “unbekannt” (unknown); type(s): not mentioned. **Pelobatesfuscusvar.lividis Koch, 1872**: type locality: “von den Wiesen in der Nähe des Röder-Wäldchens bei Frankfurt” (the meadows in the Röder groove near Frankfurt), Germany; type(s): not mentioned; ***Pelobatesinsubricus* Cornalia, 1873**; type locality: nearby Milano, Italy; type(s): not mentioned, most likely deposited at MSNM, but presumably lost since (Blackburn and Scali 2014). ***Pelobateslatifrons* Herón-Royer, 1888**; type locality: “environ de Turin” (nearby Torino), Italy; type(s): not mentioned. ***Pelobatespraefuscus* Khosatzky, 1985**; type locality: Etuliya, Moldova; holotype: ZISP 21N RNA M-1, a Pliocene fossil (according to Frost 2019). The Italian populations, for long considered as a subspecies *P.f.insubricus*, have been a matter of debate until recently because they bear private mtDNA haplotypes (Crottini et al. 2007). Litvinchuk et al. (2013) synonymized this taxon with *P.fuscus*, given the weak divergence of these haplotypes, together with the lack of differentiation of allozyme and genome content. As it stands, *P.fuscus* should thus be considered a monotypic taxon.

#### Distribution.

Widespread distribution in western, central and eastern Europe (0–810 m elevation a.s.l.), but absent from the northern European countries and most of southern Europe (Sillero et al. 2014; Nöllert at al. 2012) (Fig. 1). In the west, it reaches the eastern edge of the Netherlands (Creemers and Van Delft 2009), the eastern part of Flanders in Belgium (Bauwens and Claus 1996), the western parts of Nordrhein-Westfalens and the south-east of Rheinland-Pflaz in Germany (Bitz et al. 1996; Chmela and Kronshage 2011), the north-eastern side of France (particularly along the Rhine River, Eggert and Vacher 2012). In the north, it extends to northern Netherlands (Creemers and Van Delft 2009), the North Sea coastline of Germany (Nöllert and Günther 1996) and Denmark, the south of Sweden, as well as the coastline of the Baltic Sea from Germany to Estonia, and eastward until it reaches *P.vespertinus* in Russia (Kuzmin 1999; Nyström et al. 2007; Litvinchuk et al. 2013; Sillero et al. 2014). The contact zone with the latter is well delineated from the Kursk region in Russia to the Black Sea coast (Dufresnes et al. 2019b). From there, it is present westward along the Black Sea coast of Ukraine to north-eastern Bulgaria (Kuzmin 1999; Stojankov et al. 2011). The southern edges extend along the Danube at the borders of Romania and Bulgaria (Stojankov et al. 2011) and across Serbia (Vukov et al. 2013), eastern Croatia, northern Bosnia and Herzegovina, Slovenia (Džukić et al. 2008, Curić et al. 2018), northern and eastern Austria around the Alps (Cabela et al. 2001), and southern Germany (Nöllert and Gunther 1996). The species is also present in a large area of northern Italy, especially in the Po Valley (Andreone 2006). Last, isolated populations persist in central France (Indre, Loiret, Indre-et-Loire: Eggert and Vacher 2012) and western Bulgaria (around Sofia: Stojankov et al. 2011). IUCN Status: Not Evaluated, considered Least Concern when grouped with *P.vespertinus* (Agasyan et al. 2009a). Declines have been reported for more than a century in various parts of Europe, which have caused a regression of the distribution limits (Džukić et al. 2005; Eggert et al. 2006).

#### Diversity.

The phylogeographic work by Crottini et al. (2007) and Litvinchuk et al. (2013) characterized two refugial groups for this species (as the “western lineage of *P.fuscus*”), based on shallow mtDNA divergence and allozyme differentiation: in the Balkans/northern Italy and on the western shores of the Black Sea coast. This seems supported by weak genomic differentiation among Central-European samples (Dufresnes et al. 2019b). The refugial areas bear nearly all the genetic diversity of the species, which was lost in the derived northern populations, following post-glacial colonizations (Eggert et al. 2006).

### Pelobates vespertinus

Taxon classificationAnimaliaAnuraPelobatidae

(Pallas, 1771)

#### Diagnosis.

Morphologically close to its sister species *P.fuscus*, it similarly features pale metatarsal spades and a domed skull. The coloration also spans the gray-yellowish-brownish spectrum, including reddish individuals (Fig. 3); orange dots can be heavily marked or absent (Fig. 3). It differs from *P.fuscus* by most individuals having three light longitudinal stripes on the dorsum (Suriadna et al. 2016), as well as a dark stripe between the eyes (Lada et al. 2005). Sexes of similar size (Fig. 2). Average SVL = 47 mm (range: 29–59 mm) for females (*n* = 3 populations) and 48 mm (29–61 mm) for males (*n* = 12 populations) (Suppl. material 1; Table S1, Fig. 2). The karyotype consists of seven large and six small pairs of two-armed chromosomes (Manilo and Manuilova 2013; Suriadna 2014). NORs (secondary constrictions) are in the short arm of pair 7 (Manilo and Radchenko 2008). The nuclear DNA content averages 9.2–9.4 pg (Litvinchuk et al. 2013). As shown in Table 1, *P.vespertinus* differs from *P.fuscus* by 2.5% at mtDNA and 0.13% at nuclear DNA (Dufresnes et al. 2019b). The genome of *P.vespertinus* is about 5% larger than *P.fuscus* (Borkin et al. 2001; Litvinchuk et al. 2013; Suriadna 2014).

#### Taxonomy.

Originally named ***Ranavespertina* Pallas, 1771**; type locality: not specifically designated, but the author mentioned this taxon in Zarbay Creek (“Bach Sarbei”, Samara oblast), Russia, which can be considered as the type locality; type(s): not mentioned. Three junior synonyms. **Pelobatesfuscusvar.orientalis Severtsov, 1855**; type locality: “Voronezhskaya Gubernia” (Voronezh governorate), Russia; type(s): not mentioned. ***Pelobatescampestris* Severtsov, 1855**; type locality: between Bityug, Don and Ikorets rivers in today’s Voronezh province, Russia; type(s): not mentioned. ***Pelobatesborkini* Zagorodniuk, 2003**; proposed for the eastern form of *P.fuscus* but nomen nudum because neither a type specimen nor a type locality were designated (Zagorodniuk 2003). *Pelobatesvespertinus* was previously considered a subspecies of the common spadefoot, as *Pelobatesfuscusvespertinus* (Crochet and Dubois 2004). The significant divergence (~2–3 My) and restricted admixture with *P.fuscus*, consistent with reproductive isolation, both support the distinction of *P.vespertinus* as a separate species (Litvinchuk et al. 2013; Dufresnes et al. 2019b), as also proposed from genome size differences (Suriadna 2014).

#### Distribution.

A lowland species (0–830 m elevation a.s.l.) widespread from the contact zone with *P.fuscus*, to western Siberia and Kazakhstan, and along the Ural River (Kuzmin 1999) (Fig. 1). However, the exact limits with *P.fuscus* are not known in the northern 700 km of the distribution range. Detailed genetic data showed that the transition extends between the Kursk region to southern Ukraine (Litvinchuk et al. 2013; Dufresnes et al. 2019b). In the south, it is present along the Sea of Azov coast to the northern Caucasus (Kuzmin 1999; Suriadna et al. 2016). Spadefoot populations in the Crimea are attributed to *P.vespertinus* (Litvinchuk et al. 2013). The southernmost populations are in Dagestan, where it is sympatric with *P.syriacus* (Mazanaeva and Askenderov 2007). IUCN Status: Not Evaluated, as *P.vespertinus* was previously included in the *P.fuscus* assessment.

#### Diversity.

Crottini et al. (2007) and Litvinchuck et al. (2013) provided detailed phylogeographic accounts for this species (as the “eastern lineage of *P.fuscus*”), which consists of a homogenous clade that expanded from a single glacial refugia located in the eastern shores of the Sea of Azov. *Pelobatesvespertinus* forms a narrow hybrid zone (< 20 km) with *P.fuscus* in eastern Ukraine/western Russia (Litvinchuk et al. 2013; Dufresnes et al. 2019b).

### Pelobates syriacus

Taxon classificationAnimaliaAnuraPelobatidae

Boettger, 1889

#### Diagnosis.

Large spadefoot with whitish metatarsal spades and a flat skull. Webbing of the hind feet less developed than in *P.fuscus* and *P.vespertinus*. Sexes of similar size (Fig. 2). Coloration can be gray, yellow, greenish but rarely brown; orange dots often present, but not as abundant and marked as in some individuals of *P.fuscus*, *P.vespertinus*, or *P.balcanicus* (Fig. 4). Based on populations of *P.syriacusboettgeri*, average SVL = 68 mm (range: 40–92) for females (*n* = 5 populations) and 69 mm (57–83 mm) for males (*n* = 4 populations) (Suppl. material 1, Table S1; Fig. 2). The karyotype consists of seven large and six small pairs of two-armed chromosomes (Uğurtaş et al. 2001, from *P.s.boettgeri*). Centromeric C-bands are obvious in pairs 8 and 10 and telomeric Q-bands in the long arms of pairs 9 and 10 (Schmid 1979; Schmid et al. 1987). NORs are in the short arm of pair 7 (Schmid 1982; Schmid et al. 1987). The nuclear DNA content averages 8.2 pg (Litvinchuk et al. 2013; data from *P.s.boettgeri*).

#### Taxonomy.

Described from the Levant region as ***Pelobatessyriacus* Boettger, 1889**; type locality: “Haiffa in Syrien” (Haifa), Israel; type: SMF 1437.1a (Boettger 1892), subsequently designated as lectotype SMF 1722 (Mertens 1967). Other nomina proposed apply to *P.s.boettgeri* and *P.balcanicus* (see below).

#### Distribution.

Scattered distribution; mainly present in the Middle East with 0–2000 m elevation a.s.l. (Agasyan et al. 2009b; Uğurtas 2001; Džukić et al. 2008; Sofianidou 2012) (Fig. 1). The nominate subspecies *P.syriacussyriacus* inhabits the southern part of the distribution in the Levant, from the Syrian coast at the border of Lebanon to the southern Israeli coast, as well as in south-western Syria (Boettger 1889; Munwes et al. 2010; Sofianidou 2012). It may be extinct from western Jordan (Agasyan et al. 2009b; Disi and Amr 2010). The subspecies *P.syriacusboettgeri* occupies the remaining ranges. In the west, it is present in western Turkey and along the Aegean coastline. It also occurs in European Turkey and probably southeastern Bulgaria. Alternatively, the latter populations could belong to *P.balcanicus*, notably along the Maritsa River, and identification is pending molecular analyses. The presence of *P.syriacus* is also documented on the Greek islands of Limnos, Lesbos, and Kos (Sofianidou 2012; Strachinis and Roussos 2016). Its central distribution is poorly known and therefore not well delineated, with several isolates described in Turkey, both along the Black and Mediterranean sea coasts, as well as the central parts of Anatolia. In the northeast, *P.syriacus* reaches the southern slopes of the Caucasus, from Georgia to Azerbaijan. The northernmost records are in Dagestan, on the west coast of the Caspian Sea, where it meets *P.vespertinus* (Mazanaeva and Askenderov 2007). Further east, it is present along the southern shores of the Caspian Sea in Iran (eastern limit in Golestan; Kamali and Malekzadeh 2013). IUCN status: Not Evaluated; considered Least Concern when grouped with *P.balcanicus* (Agasyan et al. 2009b).

#### Diversity.

Using mtDNA and genomic data, Dufresnes et al. (2019b) evidenced a Pleistocene split between the Levant (*P.s.syriacus*) and the rest of the range (*P.s.boettgeri*; see below). Within both subspecies, populations are weakly differentiated despite their present-day fragmentation (see also Munwes et al. 2010 for *P.s.syriacus*). Populations from the Caucasus (*P.s.boettgeri*) differs from Anatolian ones at nuclear, but not mitochondrial markers. In the Lesser Caucasus and southern Turkey, *P.s.boettgeri* features traces of past gene flow with *P.s.syriacus.* Iranian populations have not been examined with genetic tools and could bear cryptic diversity.

### Pelobates syriacus boettgeri

Taxon classificationAnimaliaAnuraPelobatidae

Mertens, 1923

#### Diagnosis.

Similar to the nominal subspecies, notably in terms of cranial characters (Roček 1981) and coloration patterns (Fig. 4). Most biometric data on *P.syriacus* come from populations of *P.s.boettgeri* (Fig. 2, see above). As shown in Table 1, *P.s.boettgeri* differs from *P.s.syriacus* by 1.7% at mtDNA and 0.01% at nuclear DNA (Dufresnes et al. 2019b).

#### Taxonomy.

The oldest nomen available for Anatolian/Caucasian spadefoots is ***Pelobatessyriacusboettgeri* Mertens, 1923**; type locality: Belesuwar, southeastern Azerbaijan; holotype: SMF 1725 (originally 1437.2a, Mertens 1923). A single junior synonym. ***Pelobatestranscaucasicus* Delwig, 1928**; type locality: “Tiflis” (Tbilisi), Georgia; types: ten syntypes, nine at ZISP, and one at ZIK (Amph A5/A (2164)). Subspecies level of *P.s.boettgeri* is granted by its phylogenetic divergence from *P.s.syriacus*, but the recent split (~1 My) and the widespread traces of admixture between both subspecies in Armenia, Turkey (Antalya region), and Israel argue against a specific status.

#### Distribution and diversity.

See the accounts for *P.syriacus*.

### Pelobates balcanicus

Taxon classificationAnimaliaAnuraPelobatidae

Karaman, 1928

#### Diagnosis.

Resembling *P.syriacus* with which it was previously considered a synonym (Frost 2019). Large toad with whitish metatarsal spades and a flat skull. Sexes of similar size (Fig. 2). Various motifs with gray, yellow or greenish colors, but rarely brown (unlike the sympatric *P.fuscus*, P. Székely pers. comm.); frequently specked with orange dots, sometimes heavily (perhaps more than in *P.syriacus*) (Fig. 4). Based on 25 biometric characters, Uğurtas et al. (2002) showed that the *P.balcanicus* populations from the Balkans are morphologically very variable and differentiated from Asia Minor (*P.syriacus*); yet *P.syriacus* populations from European Turkey (Edirne, genetically confirmed by Dufresnes et al. 2019b) and southeastern Bulgaria (Primorsko) grouped with *P.balcanicus* (Uğurtas et al. 2002). Roček (1981) only found one cranial difference: the *processus posterior parasphenoidei* is present in *P.syriacus* but not developed in *P.balcanicus*. Morphometric assessments associated to genetic data are needed. Based on populations of *P.balcanicusbalcanicus*, average SVL = 67 mm (48–100 mm) for females (*n* = 16 populations) and 68 mm (46–94 mm) for males (*n* = 15 populations) (Suppl. material 1, Table S1; Fig. 2). The karyotype (*P.b.balcanicus*) consists of six large and seven small pairs of two-armed chromosomes. NORs (secondary constrictions) are in the short arm of pair 7 (Belcheva et al. 1977). The nuclear DNA content (calculated from flow cytometry) averages 7.9 pg (Litvinchuk et al. 2013; data from *P.b.balcanicus*). As shown in Table 1, *P.balcanicus* differs from *P.syriacus* by ~7.4% at mtDNA and ~0.31% at nuclear DNA (Dufresnes et al. 2019b).

#### Taxonomy.

Originally described as a subspecies of the Eastern spadefoot, ***Pelobatessyriacusbalcanicus* Karaman, 1928**; type locality: Dojran Lake, North Macedonia; type(s): most likely include the skeleton described by Karaman (1928), deposited at MMNH (Skopje, North Macedonia), but destroyed in an earthquake in 1963 (V. Sidorovska pers. comm.); the MMNH currently hosts one specimen from the type locality, MMNH-A-699 (collected in 2001). This taxon represents a distinct species from *P.syriacus*, due its old divergence (>6 My) and the absence of contemporary introgression at their area of contact in European Turkey, consistent with advanced reproductive isolation (Dufresnes et al. 2019b). Therefore, we herein remove *P.balcanicus* from its previous synonymy with *P.syriacus*.

#### Distribution.

*Pelobatesbalcanicus* is restricted to the Balkan Peninsula, 0–920 m a.s.l. (Džukić et al. 2008) (Fig. 1). In the north, it is present in northern Serbia and northwestern Romania. It follows the Danube River from Serbia to the Black Sea in Romania (Székely et al. 2013; Ţeran et al. 2017). There are yet some possible gaps along the Danube (e.g. around the Iron Gate: Vukov et al. 2013; Ţeran et al. 2017). In the north-west, the Great Morova River in Serbia marks its western margin (Džukić et al. 2008). Northern ranges are currently disconnected from the southern populations (Vukov et al. 2013) of North Macedonia, eastern Albania (a single location), south-west Bulgaria (Strimon River), and Greece (Džukić et al. 2008; Mollov et al. 2006; Szabolcs and Mizsei 2017). In the 1980s, Sofianidou (2012) reported the species along the western coastline of the Adriatic Sea and the northern coastline of the Gulf of Corinth (Greece), but there is no recent observation in this region. Elsewhere in Greece, it is present in Peloponnese (*P.balcanicuschloeae* ssp. nov., see below), in the eastern parts of the mainland, and along the Aegean Sea shores, from Sterea Ellas to the Evros River, until it reaches *P.syriacus* in Thrace (Džukić et al. 2008; Sofianidou, 2012). The spadefoots known from the Maritsa (Evros) River in southern Bulgaria, and along the western coasts of the Black Sea, may correspond to *P.syriacus* (Stojanov et al. 2011; Dufresnes et al. 2019b). IUCN status: Not Evaluated; previously included in *P.syriacus* assessment.

#### Diversity.

Using mtDNA and genomic data, Dufresnes et al. (2019b) evidenced a Pleistocene split (~2 My) for spadefoots from the Peloponnese (*P.balcanicuschloeae* ssp. nov.). In the rest of the range, at least three glacial lineages (<1 My) were identified: a first one in the eastern ranges, from the Carpathians to the Black Sea and as south as Greek Thrace; a second one in western ranges from Serbia to northern Greece; and a third one on the coastal island of Evia (north-east of Peloponnese). The eastern and western lineages widely admix. Populations from central Greece are yet to be examined.

### Pelobates balcanicus chloeae ssp. nov.

Taxon classificationAnimaliaAnuraPelobatidae

http://zoobank.org/A1C08645-8307-49EF-A2EB-7F09D7BCC89D

#### Type locality.

**Figure 5.**
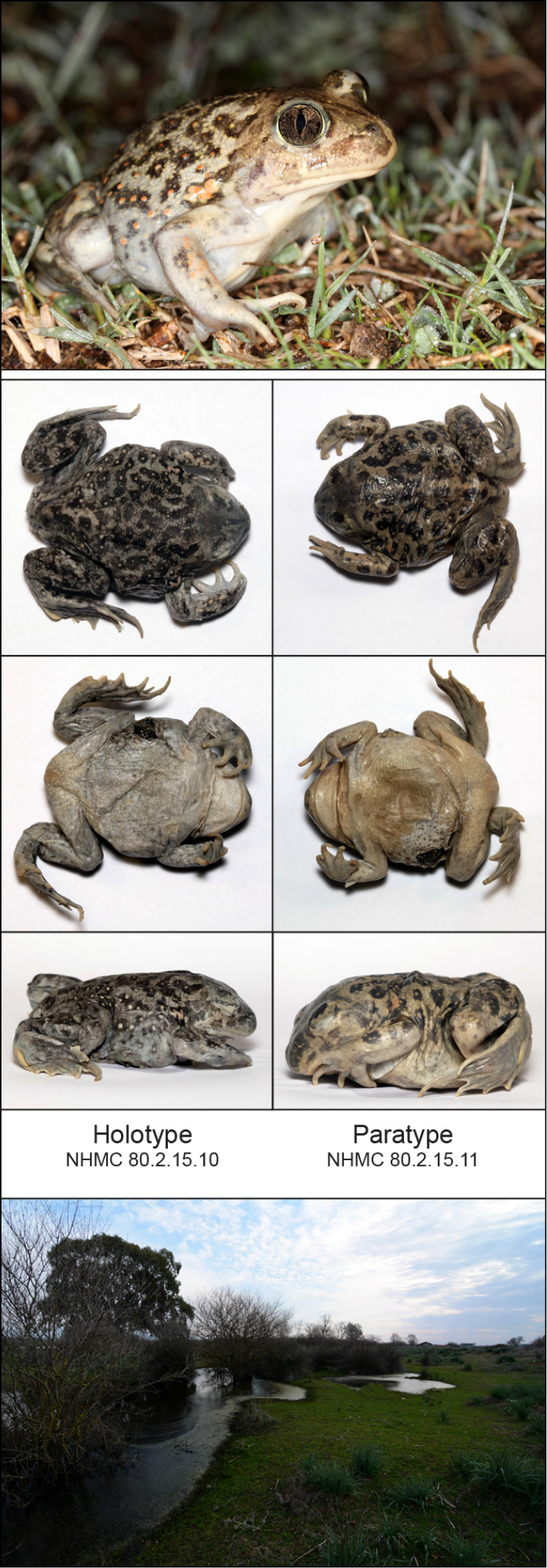
Description of *Pelobatesbalcanicuschloeae*. **Top** live photograph of the holotype, NHMC 80.2.15.10 (CD, taken on December 10^th^ 2018); **middle** dorsal and lateral views of the type specimens (**left** NHMC 80.2.15.10; **right** NHMC 80.2.15.11) post-mortem (IS); **bottom** Strofylia meadows, the type locality in Peloponnese, Greece (ET).

Strofylia meadows, near the village of Metochi, Peloponnese, Greece (38.1239°N, 21.3858°E, 1 m a.s.l.). Coastal sandy meadows with shallow ponds (Fig. 5).

#### Holotype.

NHMC 80.2.15.10, adult female captured on December 10^th^ 2018 by CD, IS and ET at Strofylia meadows, Greece (38.1239°N, 21.3858°E, 1 m a.s.l.); subsequently deposited at the Natural History Museum of Crete (NHMC); mitochondrial *cyt-b* haplotype BAL19 (Dufresnes et al. 2019b). Full measurements are available in Table 2 and photographs in Figure 5. Large specimen (SVL = 78.7 mm) with the head narrower than the body, ending by a rounded snout; nostrils closer to each other’s than from the eyes; forehead flat, as viewed from the side, with large interorbital; tympanum invisible; vomerine teeth present. Large, bulging eyes (7.2 mm of diameter) with vertical pupil and a dark-golden iris. Legs relatively short (92 mm), 1.2 times the size of the body. Five partially webbed toes; webbing formula: I 1-1+ II 1-2 III 1-2+ IV 3-1+ V; relative lengths from inner to outer toes: 4>3>5>2>1; large and long rounded (blade-shaped) metatarsal tubercle (“spade”), whitish; subarticular tubercles indistinct. Strong arms with four unwebbed fingers; palm tubercles visible, oval. Ventral and dorsal skins smooth, although the latter bears scattered warts. Coloration in life: ventrum glossy white, bluish near the limbs; dorsum light gray with prominent green-brown reticulated patches featuring orange dots, notably at the armpits; head darker, with a horizontal brown line running between the eyes. Changes of coloration in ethanol: dorsum less contrasted; fainted orange dots.

**Table 2.** Morphometric measurements (mm) of *Pelobatesbalcanicuschloeae* at the type locality (Strofylia meadows), based on 12 adults (both sexes combined), and detailed for the type specimens. SVL: snout-vent length; HW: head width; HL: head length; ED: eye diameter; EE: inter-eye distance; NN: inter-nostril distance; EN: eye-nostril distance; ML: metatarsal tubercle length; MH: metatarsal tubercle height; HLL: hind leg length; TTL: tibia + tarsus length.

	**Strofylia population**	**Holotype NMHC 80.2.15.10**	**Paratype NMHC 80.2.15.11**
SVL	71.5 ± 3.4	78.7	74.1
HW	23.7 ± 1.1	26.6	25.5
HL	21.8 ± 0.9	23.4	23.1
ED	7.4 ± 0.24	7.2	7.1
EE	15.9 ± 0.7	16.7	17.3
NN	4.4 ± 0.2	4.6	4.2
EN	6.0 ± 0.3	6.7	6.0
ML	6.1 ± 0.4	7.1	6.5
MH	2.6 ± 0.1	2.6	2.8
HLL	83.7 ± 3.6	92	90
TTL	64.2 ± 3.1	72	69

#### Paratype.

NHMC 80.2.15.11, adult female captured on December 10^th^ 2018 by CD, IS and ET at Strofylia meadows, Greece (38.1239°N, 21.3858°E, 1 m a.s.l.); subsequently deposited at the Natural History Museum of Crete (NHMC); mitochondrial *cyt-b* haplotype BAL20 (Dufresnes et al. 2019b). Full measurement and post-mortem pictures are provided in Table 2 and Figure 5, respectively.

#### Diagnosis.

Supposedly similar morphologically to the nominal subspecies and reliably diagnosed only by molecular data. So far studied from the type locality only (Strofylia). Like the nominal subspecies, *Pelobatesbalcanicuschloeae* is a large spadefoot with whitish metatarsal spades, a flat skull and incomplete webbing on the hind feet (Fig. 4). It also shares general characteristics of the genus, i.e. stocky built, smooth skin and vertical pupil; males bear oval protuberances on the arms, absent in females. The dorsum coloration is generally light gray, sometimes yellow, covered with dark green-brown reticulate patches and variable orange dots (Fig. 4). From our own observations, the color patterns seem to slightly differ from the nominal subspecies (Fig. 4). In *P.b.chloeae*, the green patches are small and numerous (fewer but larger patches in the nominal subspecies); dots are usually orange (more reddish in the nominal subspecies) and located inside the green patches (randomly distributed in the nominal subspecies). The ventrum and limbs are glossy and slightly bluish (rather pale whitish in the nominal subspecies). Moreover the snout of *P.b.chloeae* appears shorter and blunter than the nominal subspecies. These suspicions will need to be assessed by formal phenotypic analyses. At the type locality, the SVL of adults averaged 71.5 mm (range: 62–84; *n* = 12 individuals, both sexes combined). The mating call and the tadpole are yet to be described and diagnosed. The karyotype has not been documented. As shown in Table 1, *P.b.chloeae* differs from the nominal subspecies by 2.8% at mtDNA and 0.02% at nuclear DNA (Dufresnes et al. 2019b).

#### Taxonomic status.

Following Dufresnes et al. (2019b), we raise the population(s) from the Peloponnese as a distinct *P.balcanicus* subspecies based on nuclear and mitochondrial phylogenetic data, but refrain a specific status from current data, due to the relatively young evolutionary divergence (~2 My) and potential introgression with the nominal subspecies.

#### Etymology.

No name is available for spadefoots from the Peloponnese or Greece in general. We hence attribute it a new nomen, *Pelobatesbalcanicuschloeae*, as a reference to the young daughter of CD, Chloé, who played a decisive role in guiding his research towards European biogeography and herpetology. Moreover, “Chloé” is an ancient Greek name (“Χλόη”) designating the young green grass spurring from the ground in spring, reminiscent of spadefoots unearthing themselves to breed in mass. The name is also associated with Dimitra (Δήμητρα), the Ancient Greek goddess of agriculture who protected traditional farmlands in which so many amphibians used to thrive.

#### Distribution.

From current knowledge, this subspecies is endemic to the Peloponnese in southern Greece (Dufresnes et al. 2019b) (Fig. 1); it was so far genetically confirmed from its type locality only. Historically (1980s), there were records of spadefoots all over the Peloponnese, except in the three southern peninsulas (Böhme 1975; Eiselt 1988; Sofianidou 2012). Nowadays, the two known *Pelobates* localities are restricted to the central (Tripoli) and north-western (Strofylia) areas. Consequently, it is likely that there are only few populations left for this subspecies. It is not excluded that its range extends to Central Greece, where potential populations have not been examined; one sample from Kallithea Elassonos (Thessaly, Greece) bore trace of introgression by *P.b.chloeae*, suggesting past or present contact (Dufresnes et al. 2019b).

#### Ecology.

Never studied as such, but this subspecies most likely shares a similar ecology as the nominal subspecies (*P.b.balcanicus*). Inhabits open, flat, lowland areas with soft sandy soil near shallow ponds or ditches with aquatic vegetation for breeding, as described for *P.balcanicus* (Dufresnes 2019). Mostly nocturnal and semi-fossorial: comes out of the ground for foraging and breeding during / right after heavy rains. Hence it can be observed in high numbers during winter-spring showers; ET counted >70 individuals (mostly juveniles) in 15 min of search in late-October 2018 at the type locality; usually active around 13–20 °C, but also as low as 7 °C (ET pers. obs.).

#### Diversity.

Our *P.b.chloeae* samples featured the lowest nuclear genetic diversity recorded across the entire ranges of *P.balcanicus* and *P.syriacus* (Dufresnes et al. 2019b). This implies that the Strofylia population and perhaps the subspecies as a whole have been heavily bottlenecked. Two mtDNA haplotypes co-occur (Dufresnes et al. 2019b). Genetic studies are urgently needed to assess the range and diversity of this regional endemic.

**Conservation Status** – Ioannidis and Mebert (2011) mentioned road casualties at the type locality of this taxon, one of few extant populations. Although not evaluated yet, this taxon is clearly threatened according to IUCN criteria; given the narrow extent of occurrence (EOO), it should be listed as Critically Endangered (CR).

##### Identification key

Based on our updated overview of the taxonomy and distribution of *Pelobates*, we hereby provide a key to summarize the main discriminating features within this group. Because several taxa are cryptic and lack diagnostic phenotypic differences, geographic origin remains an essential information.

**Table:** 

1	Black spades on the hind legs	**2**
–	Spades of light coloration	**3**
2	Large body (6–9 cm) without orange dots, spades entirely black; Spain, Portugal and southern France	*** P. cultripes ***
–	Small body (<6 cm) with orange dots, spades bordered with black; Morocco	*** P. varaldii ***
3	Domed skull, developed webbing, and small body (<6 cm)	**4**
–	Flat skull, partial webbing, and large body (6–8 cm)	**5**
4	Dorsum stripes rare; Central and northwestern Europe, west of a Crimea–Moscow imaginary line	*** P. fuscus ***
–	Three dorsum stripes often present; Eastern Europe and Central Asia, east of a Crimea–Moscow imaginary line	*** P. vespertinus ***
5	Levantine region (Israel, Lebanon, and Syria)	*** P. syriacus syriacus ***
–	Caucasus and Caspian Sea shores, Anatolia, and European Turkey	*** P. syriacus boettgeri ***
–	Balkan Peninsula, except Peloponnese	*** P. balcanicus balcanicus ***
–	Peloponnese	*** P. balcanicus chloeae ***

## Conclusions

Our phylogeographic analyses of *Pelobates* (Dufresnes et al. 2019b) called for a taxonomic reassessment of this threatened amphibian group. We reviewed the evidence for distinct Moroccan (*P.varaldii*), Iberian (*P.cultripes*), Central (*P.fuscus*), and Eastern European (*P.vespertinus*) species. Furthermore, we revised the taxonomy of *P.syriacus* by distinguishing two cryptic species, *P.syriacus* and *P.balcanicus*, and by considering their strong intraspecific diversity into subspecific divisions, *P.s.syriacus*, *P.s.boettgeri*, *P.b.balcanicus*, and *P.b.chloeae*, the latter as a newly described taxon. Their variation in size and coloration are detailed and illustrated, based on a literature review and high-quality photographs, respectively. Finally, our paper provides up-to-date whole-range distribution maps for all extant *Pelobates* taxa.

## Supplementary Material

XML Treatment for Pelobates cultripes

XML Treatment for Pelobates varaldii

XML Treatment for Pelobates fuscus

XML Treatment for Pelobates vespertinus

XML Treatment for Pelobates syriacus

XML Treatment for Pelobates syriacus boettgeri

XML Treatment for Pelobates balcanicus

XML Treatment for Pelobates balcanicus chloeae

## Supplementary materials

Supplementary material 1 Average snout-vent length in *Pelobates* populations Data type: measurement This dataset is made available under the Open Database License (http://opendatacommons.org/licenses/odbl/1.0/). The Open Database License (ODbL) is a license agreement intended to allow users to freely share, modify, and use this Dataset while maintaining this same freedom for others, provided that the original source and author(s) are credited.Christophe Dufresnes, Ilias Strachinis, Elias Tzoras, Spartak N. Litvinchuk, Mathieu Denoël
