# Supplementary material for: Call a spade a spade: taxonomy and distribution of Pelobates, with description of a new Balkan endemic
Source: Zookeys. 2019 Jul 2;859:131–58. doi: 10.3897/zookeys.859.33634 (PMC6616056; doi:10.3897/zookeys.859.33634)
Supplement: Supplementary material 1 [file zookeys-859-131-s001.pdf]

Table S1: Average individual snout-vent length (SVL) in *Pelobates* populations (in mm).

Taxon	Locality	Female			Male			Reference
		n	mean ± SE	Range	n	mean ± SE	Range	
b. balcanicus	Utrine, Serbia	30	60.8 ± 1.7	47.8–79.6	21	61.1 ± 2.9	47.6–75.6	Rot-Nikcevic et al. 2001
b. balcanicus	Deliblato sand, Serbia	32	60.6 ± 1.5	47.8–79.6	29	60.4 ± 1.4	47.6–75.6	Uğurtas et al. 2002
b. balcanicus	Ivanovo, Serbia	9	57.1 ± 1.8	48.1–66.2	4	53.9 ± 4.4	46.1–62.1	Uğurtas et al. 2002
b. balcanicus	Staro Laniste, Serbia	2	71.0 ± 0.9	70.2–71.9				Uğurtas et al. 2002
b. balcanicus	Macedonia + Bulgaria	11	70.5 ± 1.2	66.1–79.3	19	75.8 ± 0.7	68.8–79.7	Rot-Nikcevic et al. 2001
b. balcanicus	Ovce pole, Macedonia	3	72.3 ± 2.0	68.4–75.2	4	71.5 ± 1.9	66.6–75.5	Uğurtas et al. 2002
b. balcanicus	Monospitovo, Macedonia	24	63.4 ± 1.6	54.2–83.8	15	60.5 ± 1.4	53.7–76.1	Uğurtas et al. 2002
b. balcanicus	Prdejci, Macedonia	6	70.2 ± 1.0	67.7–73.2	7	71.6 ± 3.4	59.1–85.4	Uğurtas et al. 2002
b. balcanicus	Djavato, Macedonia	13	71.5 ± 2.1	59.8–84.7	10	76.5 ± 1.6	70.0–88.2	Uğurtas et al. 2002
b. balcanicus	Ezerani, Macedonia	13	59.9 ± 1.0	52.1–68.3	11	60.6 ± 1.2	53.3–65.9	Uğurtas et al. 2002
b. balcanicus	Romania	5	73.0	67.0–79.0				Bacescu 1954
b. balcanicus	Kresna, Bulgaria	4	72.9 ± 3.0	66.1–79.3	14	76.9 ± 0.6	72.4–79.7	Uğurtas et al. 2002
b. balcanicus	Grindul Lupilor, Romania	39	74.4 ± 1.9	55.4–99.5	37	76.5 ± 1.8	59.3–93.7	Cogalniceanu et al. 2014
b. balcanicus	Durunkulak, Bulgaria	13	69.6 ± 1.5	62.8–79.4	17	67.8 ± 1.4	57.8–79.5	Uğurtas et al. 2002
b. balcanicus	Plovdiv, Bulgaria				1	90.0		Müller 1932
b. balcanicus	Greece	44	73.2	66.0–84.0	38	72.4	65.0–85.0	Sofianidou 2012
b. balcanicus	Greece	35	53.9	50.0–60.0	29	50.8	49.0–55.0	Sofianidou 2012
cultripes	Spain	?	78.0		?	70.1		Salvador et al. 1986
cultripes	Arriça, Portugal	40	54.2	?-72	20	52.8	?-62	Leclair et al. 2005
cultripes	Porto Covo, Portugal	4	72.7	?-84	11	71.5	?-80	Talavera 1990
cultripes	Montalvos, Spain	66	74.6	?-86	76	72.0	?-85	Lizana et al. 1994
cultripes	Madrid, Spain	14	79.5	?-101	15	79.8	?-87	Talavera 1990
cultripes	Cadiz, Spain	30	72.1	?-99	30	65.8	?-84	Beukema et al. 2013
cultripes	Pedroso, Spain	18	90.4 ± 2.0		56	83.9 ± 1.1		Marangoni and Tejedo 2008
cultripes	Navas, Spain	5	91.8 ± 3.8		6	83.8 ± 3.5		Marangoni and Tejedo 2008
cultripes	Gerena, Spain				8	93.3 ± 3.0		Marangoni and Tejedo 2008
cultripes	Aznalcóllar, Spain	14	104.7 ± 2.3		31	88.1 ± 1.5		Marangoni and Tejedo 2008
cultripes	Lázaro, Spain	1	68.0		18	58.4 ± 2.0		Marangoni and Tejedo 2008
cultripes	Bodogones, Spain	40	76.2 ± 1.3		43	71.6 ± 1.3		Marangoni and Tejedo 2008
cultripes	Abalario, Spain	69	71.8 ± 1.0		83	67.7 ± 0.9		Marangoni and Tejedo 2008
cultripes	Doñana, Spain	24	62.9 ± 1.7		47	66.1 ± 1.2		Marangoni and Tejedo 2008
cultripes	Doñana, Spain	8	60.8		8	55.9		Díaz-Paniagua 2005
cultripes	Mas de Melons, Spain	26	76.1		27	71.8		Pascual-Pons et al. 2017
cultripes	Spain?	12	44.9 ± 2.4	32.0–57.1	17	47.7 ± 2.6	33.9–64.3	Busack et al. 1985
fuscus	Stagni di Belangero, Italy	2	52.3 ± 0.3		12	45.6 ± 0.7		Mercurio and Li Vigni 2007
fuscus	Arsago-Seprio, Italy	26	52.1 ± 1.1	43.0–61.5	17	50.4 ± 1.6	44.5–57.5	Scali and Gentilli 2003
fuscus	Ivrea, Italy	73	54.5 ± 3.7		70	45.7 ± 2.6		Andreone et al. 2004
fuscus	Ivrea II, Italy	11	58.4 ± 2.6	55.0–63.0	22	50.2 ± 4.2	40.0–58.0	Andreone and Pavignano 1988
fuscus	Varese, Italy	?	52.1 ± 5.4		?	50.4 ± 3.4		Andreone et al. 2004
fuscus	Wien, Austria	199	45.0 ± 0.4		427	39.6 ± 0.2		Wiener 1997
fuscus	Braunschweig, Germany	289	49.7		866	43.3		Tobias 2000
fuscus	Mecklenburg, Germany	37	52.5 ± 0.9	40.0–67.0	61	46.0 ± 0.6	37.0–56.0	Szekely and Nemec 2002
fuscus	Brandeburg, Germany	25	52.6	48.0–59.0	100	42.2	40.0–48.0	Szekely and Nemec 2002
fuscus	Niedersachsen, Germany	223	63.0 ± 0.3	48.0–78.0	545	52.7 ± 0.2	38.0–65.0	Szekely and Nemec 2002
fuscus	Sachen-Anhalt, Germany	21	54.8 ± 1.2	46.0–62.0	29	47.8 ± 0.6	42.0–57.0	Szekely and Nemec 2002
fuscus	Unteren Saaletal, Germany	40	56.9 ± 0.8	48.3–69.5	65	47.0 ± 0.8	38.9–55.6	Grosse 2008
fuscus	Nordrhein-Westfalen, Germany	?		?–71.5	?		?–62.3	Chmela and Kronshage 2011
fuscus	Nordrhein-Westfalen, Germany	2	69.5 ± 1.5	68.0–71.0	7	55.7 ± 1.3	50.0–61.0	Szekely and Nemec, 2002
fuscus	Germany + Hungary	23	44.2 ± 4.0		12	42.6 ± 3.8		Andreone et al. 1993
fuscus	Aleksandrow Kujawski, Poland	157	50.3 ± 0.3	43.0–57.6	161	44.4 ± 0.2	38.0–50.9	Adnrzejewski et al. 1977
fuscus	Poland	?	?	50.0–77.0	?	?	45.0–65.0	Juszczyk 1974
fuscus	Utrine, Serbia	26	53.7 ± 1.0	46.5–65.2	5	44.8 ± 1.2	41.2–48.1	Rot-Nikcevic et al. 2001
fuscus	Cavolj, Serbia	28	54.0 ± 0.8	47.1–62.2	39	46.0 ± 0.5	40.6–56.7	Rot-Nikcevic et al. 2001
fuscus	Lesino Kopovo, Serbia	27	45.4 ± 1.8	41.4–57.9	16	43.9 ± 0.8	36.4–49.5	Rot-Nikcevic et al. 2001
fuscus	Cluj-Napoca, Romania	15	64.1 ± 1.4	52.0–72.0	80	52.2 ± 0.4	48.0–63.0	Szekely and Nemec 2002
fuscus	Cluj-Napoca, Romania	41	62.4 ± 0.8	50.0–73.0	117	54.3 ± 0.3	45.0–63.0	Szekely and Nemec 2002
fuscus	Grindul Lupilor, Romania	34	47.7 ± 1.1	36.9–61.5	43	41.7 ± 0.4	37.6–47.3	Cogalnicanu et al. 2014
syriacus boettgeri	Edirne, Turkey	5	69.4 ± 2.0	61.7–73.4	21	68.6 ± 1.1	56.7–77.6	Uğurtas et al. 2002
syriacus boettgeri	Izmir, Turkey	9	62.2 ± 4.3	40.0–79.0	12	70.8 ± 2.0	57.0–79.0	Basoglu and Zaloglu 1964
syriacus boettgeri	Izmir, Turkey	20		64.0–92.0	13		66.0–72.0	Caydam 1974
syriacus boettgeri	Seydisehir, Turkey	9	58.2 ± 1.4	50.8–63.7	13	63.1 ± 1.2	57.2–70.8	Uğurtas et al. 2002
syriacus boettgeri	Tbilisi, Georgia	9	71.5	64.4–86.0	16	71.6	61.5–82.5	Delwig 1928
syriacus boettgeri	Belesuwar, Azerbaijan	1	78.0					Mertens 1923
s. syriacus	Haifa, Israel	1	78.0		1	73.5		Boettger 1889
varaldii	Morocco	9	50.7 ± 2.7	35.6–62.5	20	51.0 ± 1.6	33.2–64.6	Busack et al. 1985
varaldii	Foret Mamora, Morocco	20	52.8 ± 1.3	45.0–64.1	66	51.7 ± 0.4	43.9–60.6	Guarino et al. 2011
varaldii	Foret Mamora, Morocco	30	53.6	?-66	98	51.9	?-61	Beukema et al. 2013
varaldii	Foret Mamora, Morocco				1	49.5		Pasteur and Bons 1959
varaldii	Merja Samora, Morocco	1	55.0					Pasteur and Bons 1959
vespertinus	Karadagh NR, Russia				3	52.9 ± 4.8	44.5–61.3	Kukushkin 2004
vespertinus	Karadagh NR, Russia				5	56.3 ± 2.2	51.3–61.4	Kukushkin 2006
vespertinus	Opuk NR, Russia				1	58.2		Kukushkin and Moroz 2007
vespertinus	Mordovia Republic, Russia	7	52.7 ± 1.5	48.1–58.0	12	42.1 ± 1.6	31.0–47.0	Ruchin 2014
vespertinus	Uritskoe, Russia	32	45.7 ± 0.7	37.8–55.1				Yermokhin et al. 2014
vespertinus	Uritskoe, Russia	?	40.0-45.4	29.3–59.2	?	35.7-43.2	29.0–56.0	Yermokhin et al. 2016
vespertinus	Uritskoe, Russia				15	48.0 ± 0.7	43.0–52.3	Yermokhin and Tabachishin 2011
vespertinus	Uritskoe, Russia				49	46.5 ± 0.5	39.7–54.8	Yermokhin and Tabachishin 2011
vespertinus	Uritskoe, Russia				21	48.9 ± 0.6	42.7–54.3	Yermokhin and Tabachishin 2011
vespertinus	Uritskoe, Russia				17	46.3 ± 0.8	40.2–52.5	Yermokhin and Tabachishin 2011
vespertinus	Uritskoe, Russia				18	46.1 ± 0.8	39.8–53.8	Yermokhin and Tabachishin 2011
vespertinus	Uritskoe, Russia				35	47.9 ± 0.5	41.3–53.1	Yermokhin and Tabachishin 2011
vespertinus	Uritskoe, Russia				15	46.4 ± 0.9	42.0–53.6	Yermokhin and Tabachishin 2011

Cited references

Andreone E, Fortina R, Chiminello A (1993) Natural history, ecology and conservation of the Italian spadefoot toad, *Pelobates fuscus insubricus*. Scientific Reports of the Zoological Society "La Torbiera" 2: 1–96.

Andreone E, Pavignano I (1988) Observations on the breeding migration of *Pelobates fuscus insubricus* Cornalia 1873 at a ditch in north western Italy (Amphibia, Anura, Pelobatidae). Bollettino della Museo Regionale di Scienze Naturali, Torino 1: 241–250.

Andreone F, Bergo PA, Bovero S, Gazzaniga E (2004) On the edge of extinction? The spadefoot *Pelobates fuscus insubricus* in the Po Plain, and a glimpse at its conservation biology. Italian Journal of Zoology 71: 61–72.

Andrzejewski H, Przystalski A, Wilczynska B (1977) The biometric structure of the common spadefoot toad (*Pelobates fuscus* Laur., Salientia, Amphibia) of the environs of Aleksandrow Kujawski Poland. Acta Biologica Cracoviensia Series Zoologia 20: 65–74.

Bacescu M (1954) *Pelobates syriacus balcanicus* Karaman, a new frog for the fauna of Romania (in Romanian). Comunicări ale Academiei Republicii Populare Romîne 4: 483–490.

Basoglu M, Zaloglu S (1964). Morphological and osteological studies in *Pelobates syriacus* from Izmir region, Western Anatolia. Senckenbergiana biologica 45: 233–242.

Beukema W, De Pous P, Donaire-Barroso D, Bogaerts S, Garci-Port J, Escoriza D, Arribas OJ, El Mouden EH, Carranza S (2013) Review of the systematics, distribution, biogeography and natural history of Moroccan amphibians. Zootaxa 3661: 1–60.

Boettger O (1889). Ein neuer Pelobates aus Syrien. Zoologischer Anzeiger 12: 144–147.

Busack SD, Maxson LR, Wilson MA (1985) *Pelobates varaldii* (Anura: Pelobatidae): A morphologically conservative species. Copeia 1985: 107–112.

Caydam, O. 1974. Izmir'de bulunan anura türlerinden *Bufo bufo*, *Bufo viridis* (Bufonidae), *Rana ridibunda* (Ranidae), *Pelobates syriacus* (Pelobatidae) ve *Hyla arborea* (Hylidae)'nin üreme biyolojisi üzerinde aras xtırmalar. Ege Universitesi Fen Fakültesi Ilmi Raporlar Serisi 198: 1–22.

Chmela C, Kronshage A (2011) Knoblauchkröte - *Pelobates fuscus*. In: Hachtel M, Schlüpmann M, Weddeling K, Thiesmeier B, Geiger A, Willigalla C (Eds) Handbuch der Amphibien und Reptilien Nordrhein-Westfalens Band 1. Laurenti Verlag, Bielefeld, 896 pp.

Cogălniceanu D, Rosioru DM, Széleky P, Székely D, Buhaciuc-Ionita E, Stanescu F, Miaud C (2014) Age and body size in populations of two syntopic spadefoot toads (Genus *Pelobates*) at the limit of their ranges. Journal of Herpetology 48: 537–545.

Delwig W (1928). Eine neue Art der Gattung *Pelobates* Wagl. aus dem zentralen Transkaukasus. Zoologischer Anzeiger 75: 24–31.

Díaz-Paniagua C, Gómez-Rodríguez C, Portheault A, de Vries W (2005) Los anfibios de Doñana. Naturaleza y parques nacionales. Serie técnica. Organismo Autónomo Parques Nacionales, Madrid, 181 pp.

Yermokhin MV, Tabachishin VG (2011) Reproductive parameters of females *Pelobates fuscus* (Laurenti, 1768) as functions of size and weight characteristics. Current Studies in Herpetology, Saratov 11: 28–39.

Grosse W-R. (2008) Dynamik und Struktur einer Population der Knoblauchkröte (*Pelobates fuscus*) im Naturpark Unteres Saaletal bei Zaschwitz. RANA 5: 141–160.

Guarino FM, de Pous P, Crottini A, Mezzasalma M, Andreone F (2011) Age structure and growth in a population of *Pelobates varaldii* (Anura, Pelobatidae) from northwestern Morocco. Amphibia-Reptilia 32: 550–556.

Juszczyk W (1974) Płazy i gady krajowe. Państwowe Wydawn. Naukowe, Warszawa, 721 pp.

Kukushkin OV (2004) Materials to ecology and morphology of little studied amphibian species in the Crimea — the common spadefoot toad, *Pelobates fuscus* (Laurenti, 1768) (Amphibia, Anura, Pelobatidae). In: Letopis' Prirody. Karadagskiy Prirodnyi Zapovednik. T. 20. Simpheropol, Sonat, pp. 152–174.

Kukushkin OV (2006) Materials to distribution, ecology and morphology of the common spadefoot toad, *Pelobates fuscus* (Laurenti, 1768), in southeastern coast of the Mountain Crimea. In: Letopis' Prirody. Karadagskiy Prirodnyi Zapovednik. T. 21. Simpheropol, Sonat, pp. 179–200.

Kukushkin OV, Moroz PA (2007) Materials to study of herpetofauna of Opuk Nature Reserve and adjacent territories of the Kerch Black Sea coast. In: Letopis' Prirody. Opukskiy Prirodnyi Zapovednik. Simpheropol, p. 2.

Leclair MH, Leclair JR, Gallant J (2005) Application of skeletochronology to a population of *Pelobates cultripes* (Anura: Pelobatidae) from Portugal. Journal of Herpetology 39: 199–207.

Lizana M, Marquez R, Martin-Sanchez R (1994) Reproductive biology of *Pelobates cultripes* (Anura: Pelobatidae) in Central Spain. Journal of Herpetology 28: 19–27.

Marangoni F, Tejedo M (2008) Variation in body size and metamorphic traits of Iberian spadefoot toads over a short geographic distance. Journal of Zoology 275: 97–105.

Mercurio V, Li Vigni F (2007) Rediscovery of *Pelobates fuscus insubricus* in the Asti Province, north-western Italy. Acta Herpetologica 2: 1–6.

Mertens R (1923) Beiträge zur Kenntnis der Gattung Pelobates Wagler. Senckenbergiana Biologica 5: 118–128.

Müller L (1932) Beiträge zur Herpetologie der südosteuropäischen Halbinsel. 1. Herpetologisch Neues aus Bulgarien. Zoologischer Anzeiger 100: 299–309.

Pasteur G, Bons J (1959) Les batraciens du Maroc. Travaux de l'Institut scientifique Chérifien. Série Zoologie 17, Rabat, 241 pp.

Pascual-Pons M, Oromi N, Pujol-Buxo E, Fibla M, Sanuy D, Montori A (2017) Life history traits of a spadefoot toad (*Pelobates cultripes*) population from a semiarid zone in the north east of the Iberian Peninsula. Herpetological Journal 27: 57–61.

Rot-Nikcevic I, Sidorovska V, Dzukic G, Kalezic ML (2001) Sexual size dimorphism and life history traits of two European spadefoot toads (*Pelobates fuscus* and *P. syriacus*) in allopatry and sympatry. Annales (Koper), Series Historia Naturalis 11: 107–112.

Ruchin AB (2014) Ecology of amphibians and reptiles of Mordovia. Communication 1. The Pallas's spadefoot toad, *Pelobates vespertinus* (Pallas 1771). Trudy Mordovskogo Gosudarstvennogo Prirodnogo Zapovednika Imeni P.G. Smidovicha, 12: 337–349.

Salvador A, Alvarez J, Garcia C (1986) Reproductive biology of a northern population of the western spadefoot *Pelobates cultripes* (Anura, Pelobatidae). In Z. Roček (Ed) Studies in Herpetology. Societas Europaea Herpetologica, Prague, pp. 403–408.

Scali S, Gentilli A (2003) Biology aspects in a population of *Pelobates fuscus insubricus* Cornalia, 1873 (Anura: Pelobatidae). Herpetozoa 16: 51–60.

Skékely P, S Nemes (2002) Sex ratio and sexual dimorphism in a population of *Pelobates fuscus* from Transylvania, Romania. Zeitschrift für Feldherpetologie 9: 211–216.

Sofianidou T (2012) *Pelobates syriacus* (Boetger, 1899)—Syrische Schaufelkröte. In: Grossenbacher K (Ed) Handbuch der Reptilien und Amphibien Europas Bd 51 Froschlurche I. Aula, Wiebelsheim, Germany, pp. 563–620.

Talareva R (1990) Evolucion de Pelobatidos y Peloditidos (Amphibia: Anura): morfologia y desarrollo del sistema esqueletico. Unpublished Ph.D. dissertation, Universidad Complutense de Madrid, Madrid.

Tobias M (2000) Zur Populationsökologie von Knoblauchkröten (*Pelobates fuscus*) aus unterschiedlichen Agrarökosystemen. Dissertation. Technische Universität Braunschweig, 149 pp.

Uğurtas IH, Ljubusavljevic K, Sidorovska V, Kalezic ML, Džukić G. (2002). Morphological differentiation of eastern spadefoot toad (*Pelobates syriacus*) populations. Israel Journal of Zoology 48: 13–32.

Wiener K (1997) Struktur und Dynamik einer Knoblauchkrötenpopulation (*Pelobates fuscus fuscus*, Laurenti 1768) nördilch von Wien – ein Vergleich der Untersuchungsjahre 1986, 1987 und 1989 bis 1995. In: Populationsbiologie von Amphibien: Eine Langzeitstudie auf der Wiener Donauinsel, Stapfia, pp. 165–181.

Yermokhin MV, Tabachishin VG (2011) Reproductive parameters of females *Pelobates fuscus* (Laurenti, 1768) as functions of size and weight characteristics. Current Studies in Herpetology, Saratov, 11: 28–39.

Yermokhin MV, Tabachishin VG, Ivanov GA (2014) Results convergence of fecundity determination of *Pelobates fuscus* (Laurenti, 1768) by full and partitial eggs counting methods. Current Studies in Herpetology, Saratov, 14: 14–18.

Yermokhin MV, Tabachishin VG, Ivanov GA (2016) Long-term dynamics of the size-weight and sexual structure in populations of *Pelobates fuscus* (Anura, Pelobatidae) in the Medveditsa river valley (Saratov region). Current Studies in Herpetology, Saratov, 16: 113–122.
